# Blood Cell Telomere Length Is a Dynamic Feature

**DOI:** 10.1371/journal.pone.0021485

**Published:** 2011-06-24

**Authors:** Ulrika Svenson, Katarina Nordfjäll, Duncan Baird, Laureline Roger, Pia Osterman, Mai-Lis Hellenius, Göran Roos

**Affiliations:** 1 Department of Medical Biosciences, Pathology, Umeå University, Umeå, Sweden; 2 Department of Pathology, School of Medicine, Cardiff University, Heath Park, Cardiff, United Kingdom; 3 Cardiology Unit, Department of Medicine, Karolinska Institutet, Karolinska University Hospital, Stockholm, Sweden; St. Georges University of London, United Kingdom

## Abstract

There is a considerable heterogeneity in blood cell telomere length (TL) for individuals of similar age and recent studies have revealed that TL changes by time are dependent on TL at baseline. TL is partly inherited, but results from several studies indicate that e.g. life style and/or environmental factors can affect TL during life. Collectively, these studies imply that blood cell TL might fluctuate during a life time and that the actual TL at a defined time point is the result of potential regulatory mechanism(s) and environmental factors. We analyzed relative TL (RTL) in subsequent blood samples taken six months apart from 50 individuals and found significant associations between RTL changes and RTL at baseline. Individual RTL changes per month were more pronounced than the changes recorded in a previously studied population analyzed after 10 years’ follow up. The data argues for an oscillating TL pattern which levels out at longer follow up times. In a separate group of five blood donors, a marked telomere loss was demonstrated within a six month period for one donor where after TL was stabilized. PCR determined RTL changes were verified by Southern blotting and STELA (single telomere elongation length analysis). The STELA demonstrated that for the donor with a marked telomere loss, the heterogeneity of the telomere distribution decreased considerably, with a noteworthy loss of the largest telomeres. In summary, the collected data support the concept that individual blood cell telomere length is a dynamic feature and this will be important to recognize in future studies of human telomere biology.

## Introduction

Telomere attrition by increasing age is a characteristic feature of peripheral blood cells as demonstrated in a large number of studies [1]–[5]. Regarding determinants for telomere length (TL), heredity is a well recognized major component [6]–[10] and for which the paternal influence seems to be of most importance [11]–[13]. There are also numerous studies indicating that blood cell TL is associated to various diseases, examples of which are diabetes, hypertension, arteriosclerosis and cancer [14]–[21]. In general, short telomeres have been coupled to increased risk of several diseases. It has been discussed whether short TL is important for disease development, if it is a biomarker for ongoing processes leading to disease or if the disease per se, or its treatment, can cause increased telomere shortening. Regarding TL and cancer risk the data is inconsistent and both short and long TL has been coupled to increased risk [22]. It should also be noted that several published studies have been unable to show any association between TL and disease (as summarized in reference [23]). Furthermore, factors like life style and stress have been indicated to influence the blood cell TL attrition rate [24]–[25]. These data are in line with the fact that the heritable impact on TL decreases by increasing age [13], signifying the impact of micro- and/or macro-environmental factors in determining TL. What also must be taken into account is that blood cell TL measurements reflect the mean TL of a number of immune cell subpopulations. The status of the immune system, i.e. cell activity, proliferation rate, cytokine levels etc., is not constant. Hence, changes in immune system activity are likely to be reflected also in blood cell TL.

Most published population studies on blood cell TL are cross-sectional and besides a gradual telomere loss by age, a consistent finding is a large inter-individual variation. However, the variation in individual TL is reduced in the oldest old group [26]. Recent longitudinal studies, using different techniques for TL determination, have shown that individuals with longer telomeres at baseline experience the largest telomere attrition rates [27]–[30]. These findings might indicate an intrinsic mechanism for TL regulation giving priority to short telomeres. Another possibility is that longer telomeres are more susceptible than short telomeres to potential telomere noxious factors, like oxidative stress, leading to increased telomere attrition [31]. Altogether, the collected data indicate that blood cell TL regulation is a complex process influenced by a multitude of factors. We hypothesized that individual blood TL is a dynamic parameter which during periods might undergo considerable losses and during other periods show stability or even be extended. We here present data that blood cell TL indeed can change substantially within a short period of time (months) and that these changes are larger than previously reported to occur during a 10 year follow up [27], supporting the notion that blood cell TL is a dynamic feature.

## Results

**Figure 1:**
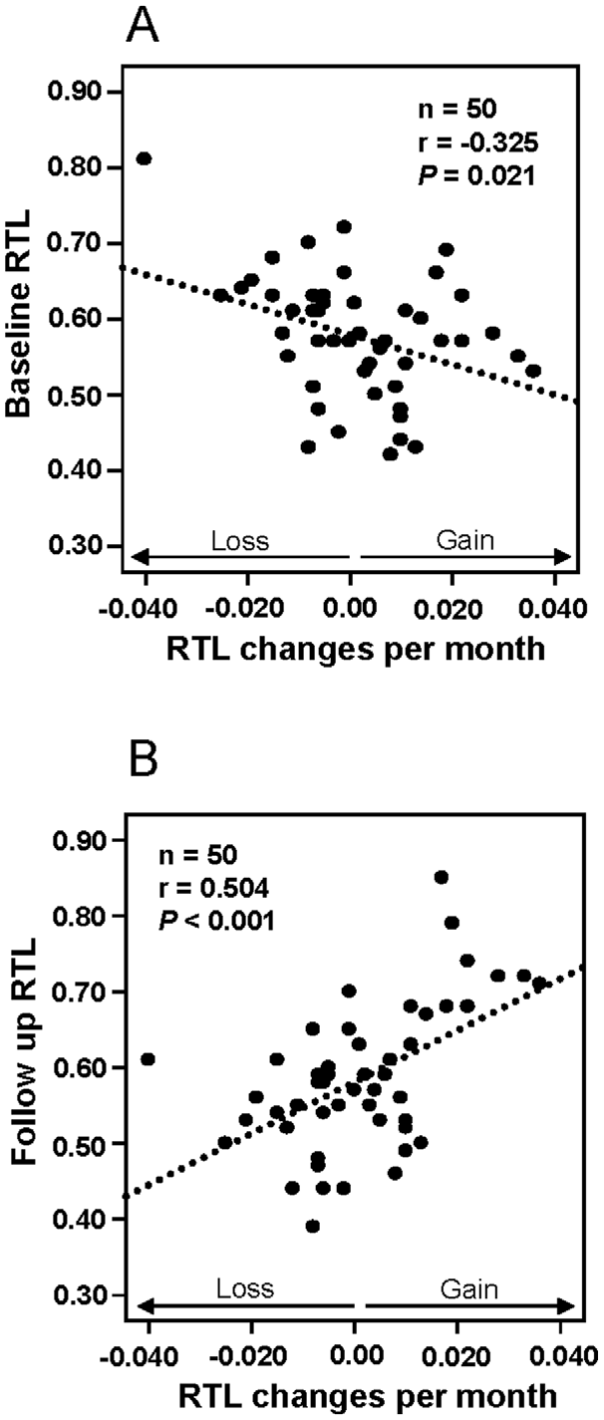
Relative telomere length (RTL) and monthly RTL changes in the 6 month study. Baseline RTL versus RTL changes per month, showing a significant negative correlation. Follow up RTL versus RTL changes per month, showing a significant positive correlation.

In the 6 month study, 50 individuals (15 men and 35 women), all of similar ages and obese, were analyzed with regard to RTL changes over time. In order to minimize the risk of methodological errors leading to a theoretical regression to the mean phenomenon, each sample was assayed twice with satisfactory reproducibility (inter-assay mean CV = 6.0%) and mean RTL values were used. There were no differences in baseline RTL (*P* = 0.356) or follow up RTL (*P* = 0.734) comparing individuals receiving physical activity on prescription (n = 25) with those receiving minimal intervention (n = 25) (Table 1). In the former group, 13 individuals showed a stable or increased RTL and 12 individuals demonstrated RTL shortening, as compared to 12 with stable/increased RTL and 13 with decreased RTL in the latter group. Since the two groups did not differ with regard to telomere length they were fused. Hence, out of the 50 individuals analyzed, 25 showed a decrease in telomere length (“RTL decreasers”) whereas 25 demonstrated elongated/stable telomeres (“RTL increasers”). RTL data for these individuals are given in table 1. At baseline, RTL decreasers had significantly longer telomeres as a group compared to the RTL increasers (*P* = 0.010; table 1). At follow up the opposite was found, i.e. RTL decreasers had significantly shorter RTL compared to RTL increasers (*P* = 0.014; table 1). There were significant correlations between RTL changes per month versus baseline RTL (fig. 1A) and follow up RTL (fig. 1B). Hence, individuals with the longest RTL at baseline tended to shorten their telomeres the most, and vice versa. No significant correlations existed between telomere length and body mass index (BMI), neither at baseline (r = 0.062; *P* = 0.667), nor at follow up ( r = −0.099, *P* = 0.495). Also, there were no significant differences in BMI comparing physical activity on prescription vs. minimal intervention groups (Baseline *P* = 0.165; Follow up *P* = 0.109), or comparing RTL decreasers vs. RTL increasers (Baseline *P* = 0.357; Follow up *P* = 0.308) (data not shown).

**Table 1:** Relative telomere length (RTL) data for individuals included in the 6 month study (median values).

	Total	Minimal intervention	Physical activity on prescription	RTL decreasers	RTL increasers
***N***	50	25	25	25	25
**Baseline RTL**	0.5776	0.5841	0.5726	0.6201	0.5602
***P*** *		0.356		0.010	
**Follow up RTL**	0.5771	0.5777	0.5765	0.5488	0.6087
***P*** *		0.734		0.014	
**RTL change/month**	−0.0005	−0.0014	0.0009	−0.0072	0.0099
***P*** *		0.308		<0.001	

Note: Age of participants: 67–68 yrs; 15 men and 35 women.

*) Between group differences (physical activity on prescription vs. minimal intervention, and RTL

decreasers vs. RTL increasers) were tested by Mann-Whitney U-tests.

**Figure 2:**
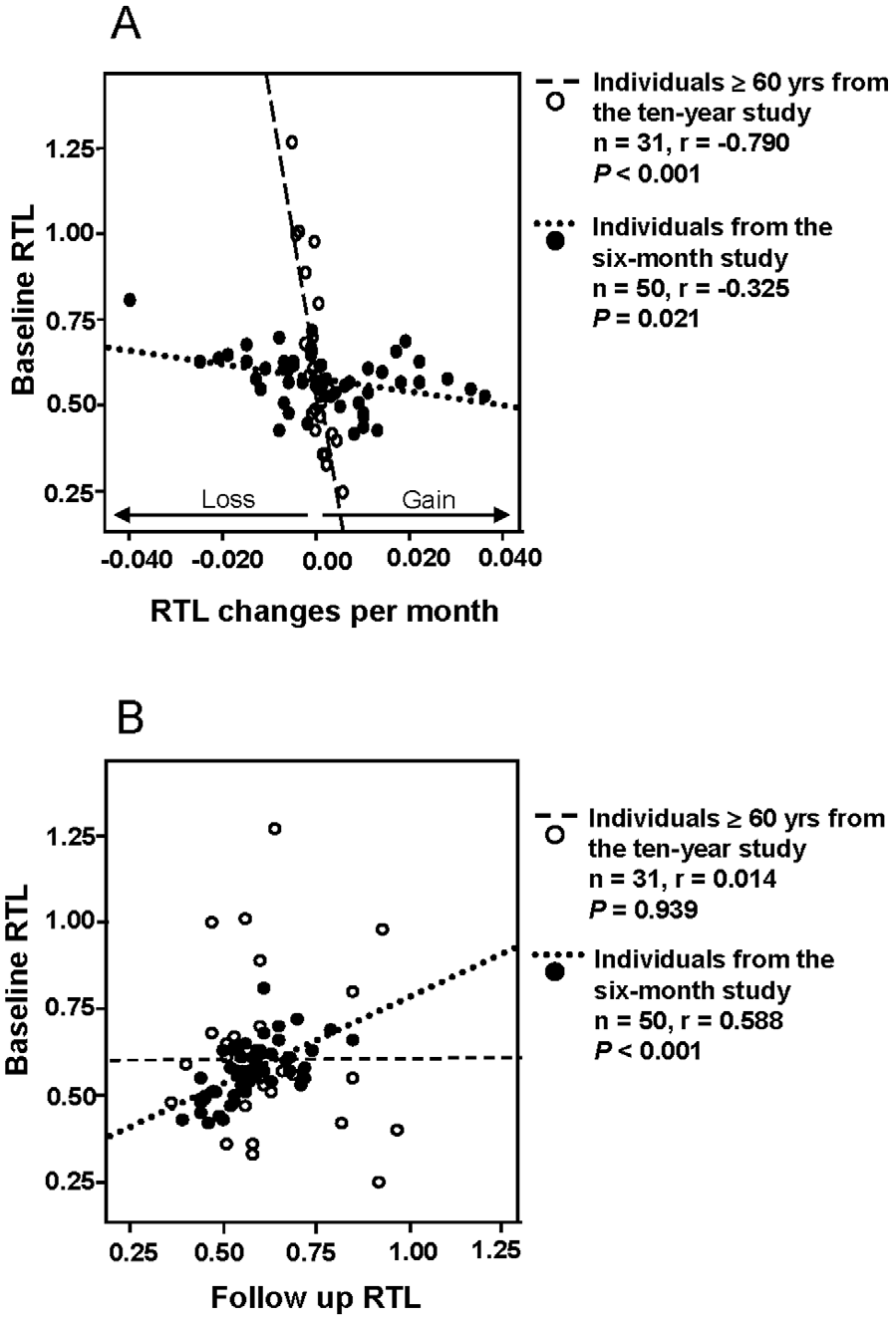
Data comparisons between the 6 month study and the 10 year study. A. Baseline RTL versus RTL changes per month. Monthly RTL changes were greater in the 6 month study compared to the 10 year study. B. Baseline RTL versus follow up RTL, showing a significant correlation in the 6 month study but not in the 10 year study.

The pattern of an association between RTL changes and RTL at baseline is in concordance with our previously published data from the 10 year study [27]. Therefore, we reanalyzed data from that study and calculated RTL changes per month (instead of per year). Only individuals ≥60 years of age at baseline were selected (n = 31) in order to better match the ages of the 6 month study participants. Monthly RTL changes in the 10 year study were found to be much smaller compared to the changes in the 6 month study. This is illustrated in figure 2A, showing data from the two studies plotted together. At the same time, there was a significant correlation between baseline samples and follow up samples in the 6 month study (r = 0.588, *P*<0.001), which was not seen in the 10 year study (r = 0.014, *P* = 0.939), as demonstrated in figure 2B.

**Figure 3:**
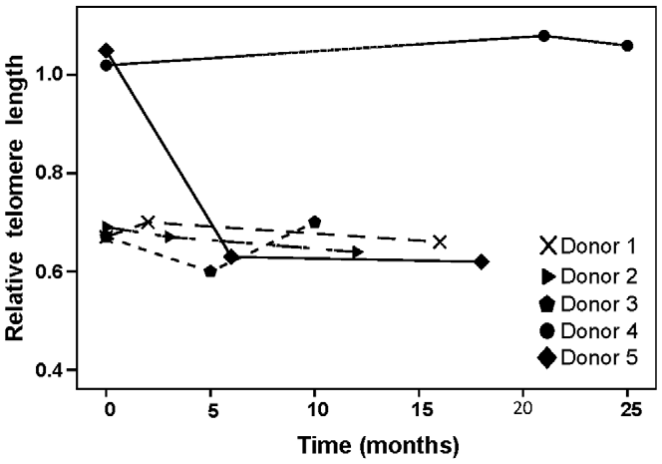
RTL values of subsequent blood samples obtained from five blood donors. Donor 5 demonstrated a marked change (decrease) in RTL between the first and second blood draw, which was not seen in any other donor.

**Figure 4:**
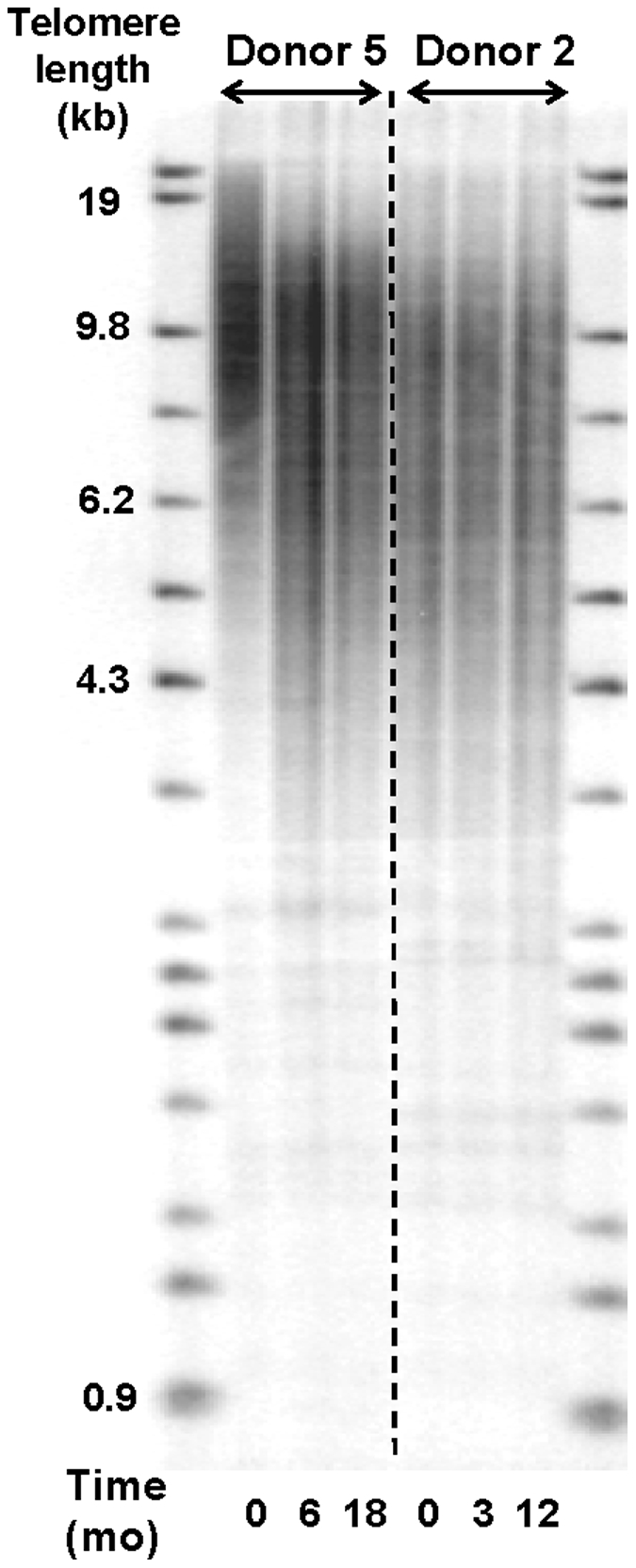
Telomere length distributions (blood donor 5 and 2) analyzed by Southern blot. The Southern blot analysis verified the RT-PCR results, showing a marked telomere length shortening for donor 5 during a six-month period. Donor 2 was used as a control.

**Figure 5:**
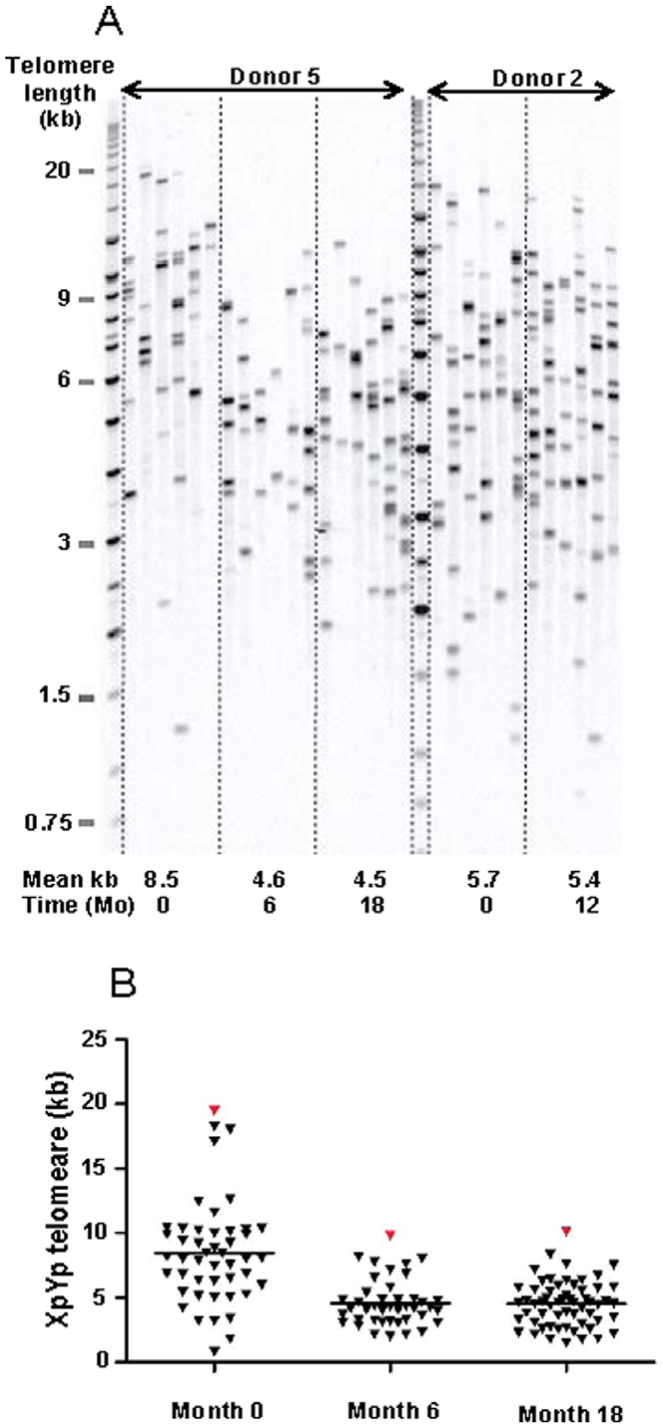
XpYp telomere end distributions in single telomere length analysis (STELA) (6 lanes/sample). A. Comparison between blood donors 5 and 2 (control), showing similar patterns as the results from RT-PCR and Southern blot. The 3 month data for Donor 2 could not be included in the STELA, due to a lack of DNA after the Southern blot analysis. B. The telomere distribution in the samples from Donor 5, showing a considerable decrease in heterogeneity and a loss of the largest telomeres.

In a separate material of five blood donors, subsequent blood samples (three samples per donor) were analyzed with regard to RTL changes over various time spans. RTL changes for each donor are demonstrated in figure 3, showing that for four of these donors RTL stayed rather stable over time, with only small fluctuations. However, one donor (Donor 5) demonstrated a large telomere loss between the first and second sample taken 6 months apart, where after RTL was stable for 12 additional months. The RTL change between the first and second sample was so noteworthy that additional, frozen samples from this donor were thawed and tested, showing the same results. To further verify this finding we performed Southern blotting, which demonstrated very similar findings to the telomere-PCR data as illustrated in figure 4, showing Donor 5 and Donor 2 (control). The result was also verified by the STELA method, analyzing the XpYp chromosome telomeres (fig. 5). Donor 2 was selected as a control since RTL remained rather stable over time, and there were sufficient amounts of DNA to perform RT-PCR, Southern Blot and STELA. For Donor 5, the mean kbp loss between sample one and two was estimated to be approximately 3.9 kbp over a 6 months period (i.e. an average monthly decrease of ∼0.65 kb) (fig. 5A). The heterogeneity of the telomere distribution was also decreased considerably, with a noteworthy loss of the largest telomeres (fig. 5B). The longest telomere end in the first sample was 19.5 kb, compared to at six months 9.8 kb and 10.1 kb at 18 months (a change of 9.7 kb). To test the concordance between the telomere-PCR and STELA method, a total of 11 DNA samples were included in the STELA (5 DNA samples from the Blood donor study shown in Fig. 5, plus 6 DNA samples from the 6 month study). The two methods correlated well with each other, with an R^2^ value of 0.79 (data not shown).

## Discussion

In the present study we show that the individual blood cell TL can experience shortening as well as elongation during a rather short period of time (months). In concordance with previously published data of longer time spans [27]–[30], there was a negative correlation between baseline RTL and RTL changes over time. As compared to our previously published data from the 10 year study [27], the monthly RTL changes were greater in the 6 month study. In individual cases remarkable TL changes could be observed, best exemplified in the present study by one of the blood donors who showed a loss of ∼3.9 kbp in the mean telomere length distribution over a 6 months period and a loss of 9.7 kb in the upper limit of telomere length. Taken together the data show that blood cell TL in individual cases can be a highly dynamic feature exhibiting considerable length changes over comparatively short periods of time.

In early life there is a strong impact of inheritance on blood cell TL [6]–[13]. The strength of the heredity factor appears however to diminish by age, and we previously showed that parent-child RTL correlations were considerably stronger at younger ages (R^2^ ∼50% at parental age<50 years) compared to older ages (R^2^<5% at parental age >70 years) [13]. The vast majority of studies investigating blood cell telomeres in various conditions or diseases have been performed on persons older than 50 years of age. Thus, the RTL values observed in those individuals are, most likely, only to a minor extent an effect of heredity and rather due to a combined effect of positive and negative mechanisms affecting blood cell TL during life.

Oxidative stress, which experimentally can induce accelerated telomere loss [31], is a common feature for a number of conditions where shorter telomeres have been found in patients compared to controls. Examples of such conditions include abnormal blood lipid levels and diabetes but also chronic psychological stress [32]–[34]. Telomere related effects of oxidative stress might be more pronounced in tissue compartments with high cellular turnover, such as the bone marrow. Since peripheral blood cells originate from the bone marrow, telomere alterations in bone marrow progenitors should be reflected also in peripheral blood cells. Indeed, it was previously shown that the telomere length of peripheral leukocytes correlated well with their bone marrow precursors in patients with coronary artery disease [35]. A long-term burden of oxidative stress, such as in chronic diseases, might be a contributing factor for RTL alterations. Interestingly, persons exposed to intense oxidative stress were recently shown to elongate the telomeres in granulocytes and naïve T cells but not in memory T and B cells. The elongation was then followed by a TL reduction [36]. These fluctuations in TL (increases and losses) occurred during 12 months observation and could theoretically be explained by a “population mixture model for nonlinear telomere dynamics” proposed by Itzkovitz et al [37]. These data and ours do not disagree with the notion that “age dependent shortening is the rule” as discussed by Chen et al [38], but elongated telomeres can in fact be recorded at certain periods of a life time, and the mechanisms behind these observations remain to be elucidated. In the present study, RTL measurements were performed two or three times at different occasions and the mean values were used for further statistics, in order to avoid measurement errors as an explanation to the data as argued by Chen et al [38]. Thus, we believe that there is a true biological variation in blood TL. Seen over a life time, telomere shortening will occur in each person, although not in a linear fashion.

The status of the immune system should also affect the estimated blood cell TL at a certain time point. For example, there is a shift from naïve T cells toward memory T-cells with ageing [39] and memory T cells have shorter telomeres than their naive counterparts [40]. The ratio between e.g. granulocytes and lymphocytes might also vary. The cellular composition of peripheral blood at a specific time point might thus be a relevant factor. Also, a less active immune system or a suppressed immune system might, at least theoretically, lead to less telomere attrition due to less cellular proliferation. In addition, molecular factors with potential positive influences on telomere length could also be of importance. For example, it is known that estrogen can activate the hTERT promoter of telomerase [41] and several cytokines have telomerase stimulatory effects [42]–[43]. We recently found that serum levels of IL-6, IL-7, IL-8 and IL-10 were positively correlated to the TL in renal cell carcinoma tumor tissue, however not to TL in blood cells in these patients (unpublished data).

**Figure 6:**
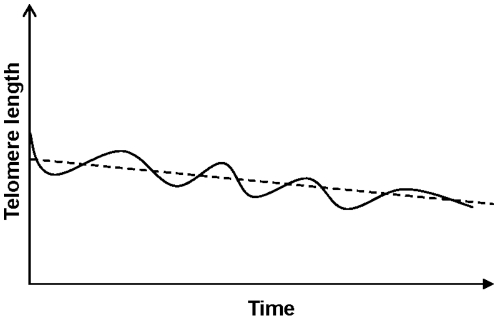
The oscillation hypothesis. Hypothetical illustration of RTL changes over time at the individual (solid line) and population (dotted line) level, based on the collected data from the present study and the literature.

Irrespective of the biological background, leukocyte telomeres appear to oscillate in length over time. We created a model, as shown in figure 6, in which blood cell TL is showing an oscillating pattern which levels out at longer follow up times**.** Future longitudinal studies are needed with shorter intervals between sampling in order to substantiate the rate by which RTL changes can occur *in vivo*. We believe that the dynamic feature of leukocyte telomeres is important to recognize in future studies of human telomere biology.

## Materials and Methods

### Ethics statement

This study was approved by the regional ethics committee in Stockholm, Sweden (2006/229-31/3 and 04-520/2).

### Samples investigated

#### The “10 year study”

Data from a previously published, population based longitudinal study on 959 individuals [27] were statistically reanalyzed regarding TL changes over time. From these individuals two blood samples had been obtained within a time span of about 10 years, thereby giving the cohort its name “the 10 year study”. The reanalysis was restricted to individuals aged 60 years and older at baseline (n = 31). Detailed information regarding sampling and TL analyses are given in reference 27.

#### The “6 month study”

56 individuals, 67–68 years of age, sedentary, overweight and with abdominal obesity were investigated for telomere length in whole blood samples taken on average 6 months apart (mean: 6 months, SD: 1,1). During the six-month period, half of the participants received physical activity on prescription whereas the other half received minimal intervention (written general information about physical activity). In the present study TL changes during the six months period were determined and data regarding effects of physical activity and other parameters have been presented elsewhere [44].

#### The “Blood donor study”

Three subsequent blood samples were obtained from five blood donors at intervals ranging from 2 to 25 months. The donor group comprised one woman and four men, with baseline ages ranging from 26 to 43 years.

### Telomere length PCR

The methodology for TL determinations has been detailed previously [11]. In short, relative telomere length (RTL) was measured using quantitative real-time PCR and calculated by the ΔΔCt method. Telomere and β-globin primer sequences written 5′ to 3′ were:

CGGTTTGTTTGGGTTTGGG-TTTGGGTTTGGGTTTGGGTT (Tel1b),

GGCTTGCCTTACCCTTACCCTTACCCTT-ACCCTTACCCT (Tel2b),

TGTGCTGG-CCCATCACTTTG (HBG3),

ACCAGCCACCACTTTCTGATAGG (HBG4).

All samples were loaded as triplicates. Telomere/single copy gene (T/S) values were calculated by 2^-ΔCt^ and relative T/S values (i.e. RTL values) were generated by dividing sample T/S values with the T/S value of a reference cell line DNA (CCRF-CEM), included in each plate. To monitor the PCR efficiency (*E*), all plates also included a standard curve. The efficiency of an RT-PCR reaction can be calculated by using the formula E = 10^[−1/slope]^ −1. Ideally the efficiency equals 100%. The mean (+/− SEM) slope-values for the telomere and HBG assays in the current study were −3.52 (+/−0.02) and −3.11 (+/−0.03) respectively, generating efficiencies of ∼92% (telomere assay) and 109% (HBG assay), which is within the recommended range [45].

In the 6 month study, a total of 112 blood samples (56 individuals, two samples per person) were analyzed. Until DNA extraction, whole blood cells were frozen and kept at −70°C. DNA extraction was performed using the BioRobot M48 Workstation with MagAttract technology as described elsewhere (Qiagen, Germany). For each DNA sample, telomere length was measured twice at different occasions. The mean inter-assay coefficient of variation (CV) for these samples was 6.7%. Six samples had a CV>15% and were excluded from further analysis. The mean CV for the remaining samples was 6.0%. Thus, TL changes were evaluated for 50 individuals. The RTL value given for each sample is the mean value of the separated PCR amplifications.

In the Blood donor study, buffy coats were separated by density gravity centrifugation (Ficoll-Hypaque; GE Healthcare). PBMC were cryopreserved in liquid nitrogen in 10% DMSO and 90% heat-inactivated FBS for later usage. Each sample was analyzed at three separate occasions (mean inter-assay CV = 5.3%). Otherwise, the PCR analysis was performed as described above.

### Southern blotting

Six DNA samples from the Blood donor study were selected for the Southern blot analysis [three from Donor 2 (control) and three from Donor 5)]. 5.5 µg DNA was cut over night at 37°C with Hinf I. The DNA was separated on a 0.6% agarose gel, 30V for 40 hours. The DNA was transferred to a Hybond- XL membrane (GE Healthcare/Amersham Biosciences, Sweden) and the membrane was air dried and UV cross linked. The membrane was prehybridized for 15 minutes at 45°C in QuikHyb solution (Stratagene, USA). A mixture of ^32^P-end labeled (T_2_AG_3_)_4_-probe (minimum 2,5×10^6^ cpm/2 ml QuickHyb solution) and salmon sperm DNA (1 mg) was added to the QuikHyb solution and hybridized to the DNA at 45°C for one hour. The membrane was washed 2×15 min in 2X SSC +0,1% SDS in room temperature and for 30 min at 45°C in 0,1X SSC +0,1% SDS. The membrane was covered with plastic and exposed to a phosphor screen over night or up to 4 days. The screen was scanned in a Typhoon 9400 imager (GE Healthcare/Amersham Biosciences, Sweden).

### Single telomere length analysis (STELA)

Six DNA samples were selected from the 6 month study (three baseline samples and three matched follow up samples) and five DNA samples from the Blood donor study (two from Donor 2 (control) and three from Donor 5), based on data from the PCR based RTL measurements. For TL analyses at the XpYp telomeres, we used a modification of the STELA assay previously described [46]–[47]. Briefly genomic DNA was solubilised by digestion with EcoRI, quantified by Hoechst 33258 fluorometry (BioRad, Hercules, USA) and diluted to 10 ng/µl in 10 mM Tris-HCl pH 7.5. 10 ng of DNA was further diluted to 250 pg/µl in a volume of 40 µl containing 1 µM Telorette2 linker and 1 mM Tris-HCl pH 7.5. Multiple PCRs (6 reactions per sample) were carried out for each test DNA in 10 µl volumes 250 pg of DNA, 0.5 µM of the telomere-adjacent and Teltail primers, 75 mM Tris-HCl pH8.8, 20 mM (NH_4_)_2_SO_4_, 0.01% Tween-20, 1.5 mM MgCl_2_, and 0.5 U of a 10∶1 mixture of Taq (ABGene, Epsom, UK) and Pwo polymerase (Roche Molecular Biochemicals, Lewes, UK). The reactions were cycled with an MJ PTC-225 thermocycler (MJ research, Watertown, USA). The DNA fragments were resolved by 0.5% TAE agarose gel electrophoresis, and detected by two separate Southern hybridisations with random-primed α-^33^P labelled (Amersham Biosciences, Little Chalfont, UK) TTAGGG repeat probe and a telomere-adjacent probe, together with a probe to detect the 1 kb (Stratagene, La Jolla, USA) and 2.5 kb (BioRad) molecular weight marker. The hybridised fragments were detected by phosphorimaging with a Molecular Dynamics Storm 860 phosphorimager (Amersham Biosciences, Little Chalfont, UK). The molecular weights of the DNA fragments were calculated using the Phoretix 1D quantifier (Nonlinear Dynamics, Newcastle-upon-Tyne, UK).

### Statistics

PASW Statistics 18.0 (SPSS Statistics) was used for statistical evaluation. Monthly RTL changes were calculated by dividing delta RTL (i.e. follow up RTL – baseline RTL) with the number of months between the dates of sample collections. Between-group differences were tested with Mann-Whitney U tests. Correlation analyses between baseline RTL, follow up RTL and RTL changes (per month) were performed using Spearman’s correlation analyses. Included in the statistical evaluation were 50 individuals from the 6 month study, 31 individuals from the 10 year study, and 5 individuals making up the Blood donor study.
